# Measurement data of an air-to-air membrane enthalpy and a heat exchanger

**DOI:** 10.1016/j.dib.2019.104453

**Published:** 2019-09-12

**Authors:** Dietmar Siegele

**Affiliations:** Unit for Energy Efficient Building, University of Innsbruck, Technikerstraße 13, AT-6020, Innsbruck, Austria

**Keywords:** Enthalpy exchanger, Heat exchanger, Ventilation

## Abstract

The data presented in this article are the measurement results of an air-to-air enthalpy exchanger and a heat exchanger. Such exchangers are used in ventilation devices. The data include 86 measurement points with different boundary conditions (winter and summer condition). The temperature, the humidity, the volume flow and all relevant pressures and pressure differences are provided. The data are mean values of a steady-state measurement. For more insight and interpretation of the results please see “Effectiveness of a membrane enthalpy heat exchanger” [1].

**Table:** Specifications Table

Subject area	*Energy and Building*
More specific subject area	*Exchanger technology, ventilation systems*
Type of data	*Table, figure*
How data was acquired	*Ventilation test rig including volume flow, temperature, humidity, pressure*
Data format	*Raw, steady-state*
Experimental factors	*Steady-state results for boundary conditions usually expected in the temperate climate zone*
Experimental features	*Measurement of all relevant parameters at the inlets and outlets; volume flow, temperature, humidity, pressure*
Data source location	*Ventilation test rig, University of Innsbruck*
Data accessibility	*Data is with this article*
Related research article	*D. Siegele and F. Ochs, “Effectiveness of a membrane enthalpy heat exchanger,” Appl. Therm. Eng., vol. 160, p. 114005,*https://doi.org/10.1016/J.APPLTHERMALENG.2019.114005*, Sep. 2019.*

**Table:** 

**Value of the Data** • The data can be used to validate models of enthalpy and/or heat exchangers with different detail levels (1D, 2D or 3D). • The boundary conditions used during the measurements can be usually expected in the temperate climate zone. With this data the influence on an overall system (i.e. a ventilation unit) using an enthalpy exchanger could be obtained. This refers to single effectiveness parameters (such as temperature, humidity, enthalpy), but also to system performance parameters (such as coefficient of performance) including the pressure drop over the heat and enthalpy exchanger. • The data set includes 104 steady-state measurement points without inter- or extrapolation. They show the full behavior of the exchangers in a wide range. Prediction of the frost risk of both types of exchangers could be carried out. By means of building simulation the impact on the indoor air quality could be shown by directly comparing heat and enthalpy exchanger. New control strategies (i.e. for humidification or de-humidification purposes) could be achieved.

## 1 Data

**Fig. 1:**
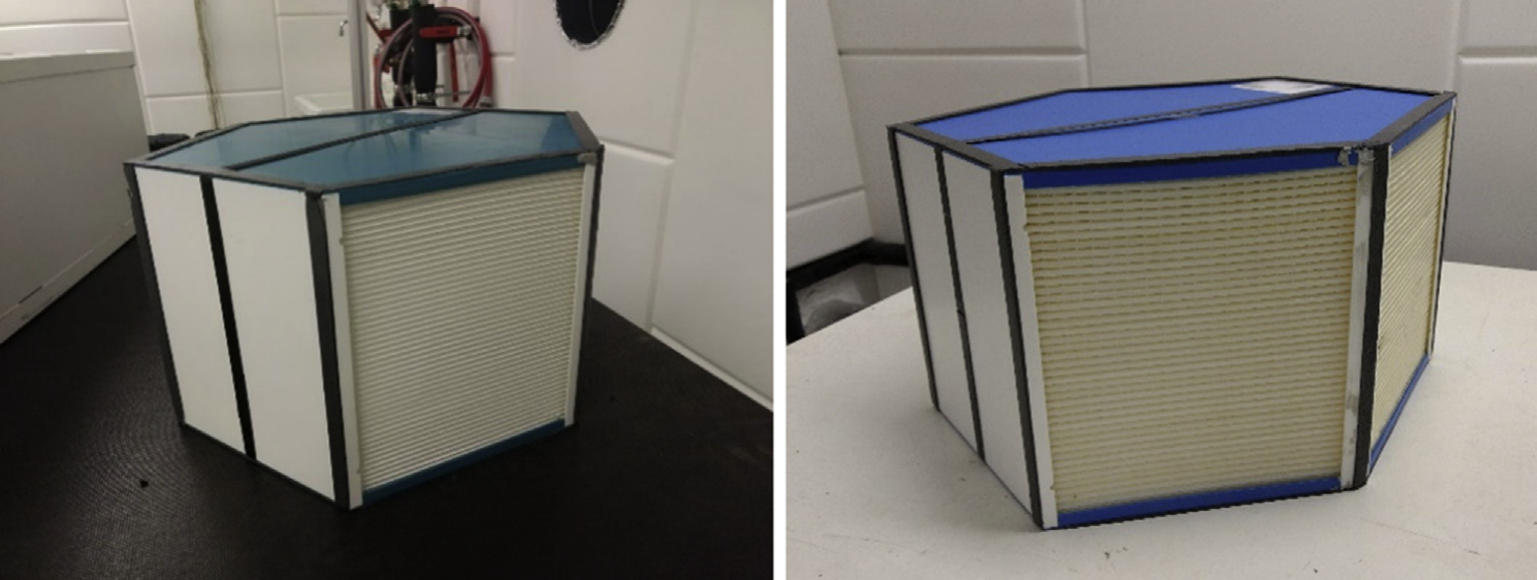
Picture of the heat exchanger (left) and enthalpy exchanger (right).

The data presented in this article were acquired in the ventilation test rig of the University of Innsbruck. The characteristics of a heat and an enthalpy exchanger, shown in Fig. 1, were measured for different boundary conditions. Altogether 104 steady-state measurement data points could be arrived, which include a temperature range between −7 °C and +30 °C for different relative humidities. The detailed boundary conditions can be found in the section Boundary conditions.

The data set includes two tables with the raw data of the steady-state results of the experiment (see section Experimental set-up for the description of the variables). The data set is separated for the heat exchanger and enthalpy exchanger. Each data point represents a steady state result that is a mean value over 30 min. For at least another 20 min before starting the measurement the standard deviation of the temperatures had to be smaller than ±0.05°C.

**Fig. 2:**
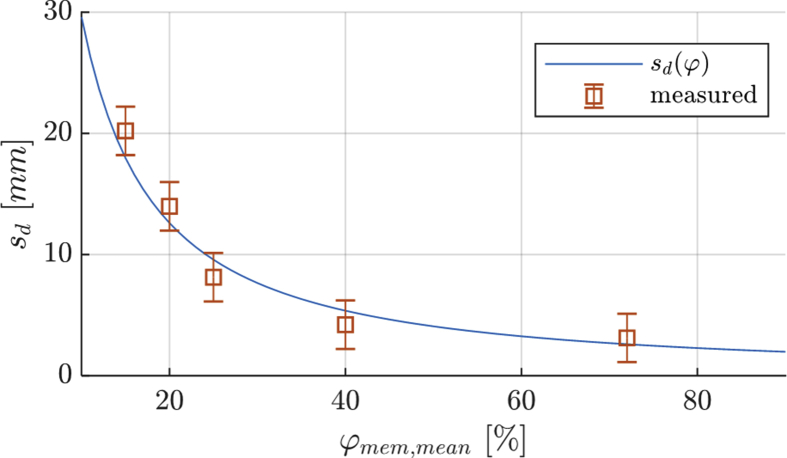
Equivalent air layer thickness of the enthalpy exchanger's membrane.

**Fig. 3:**
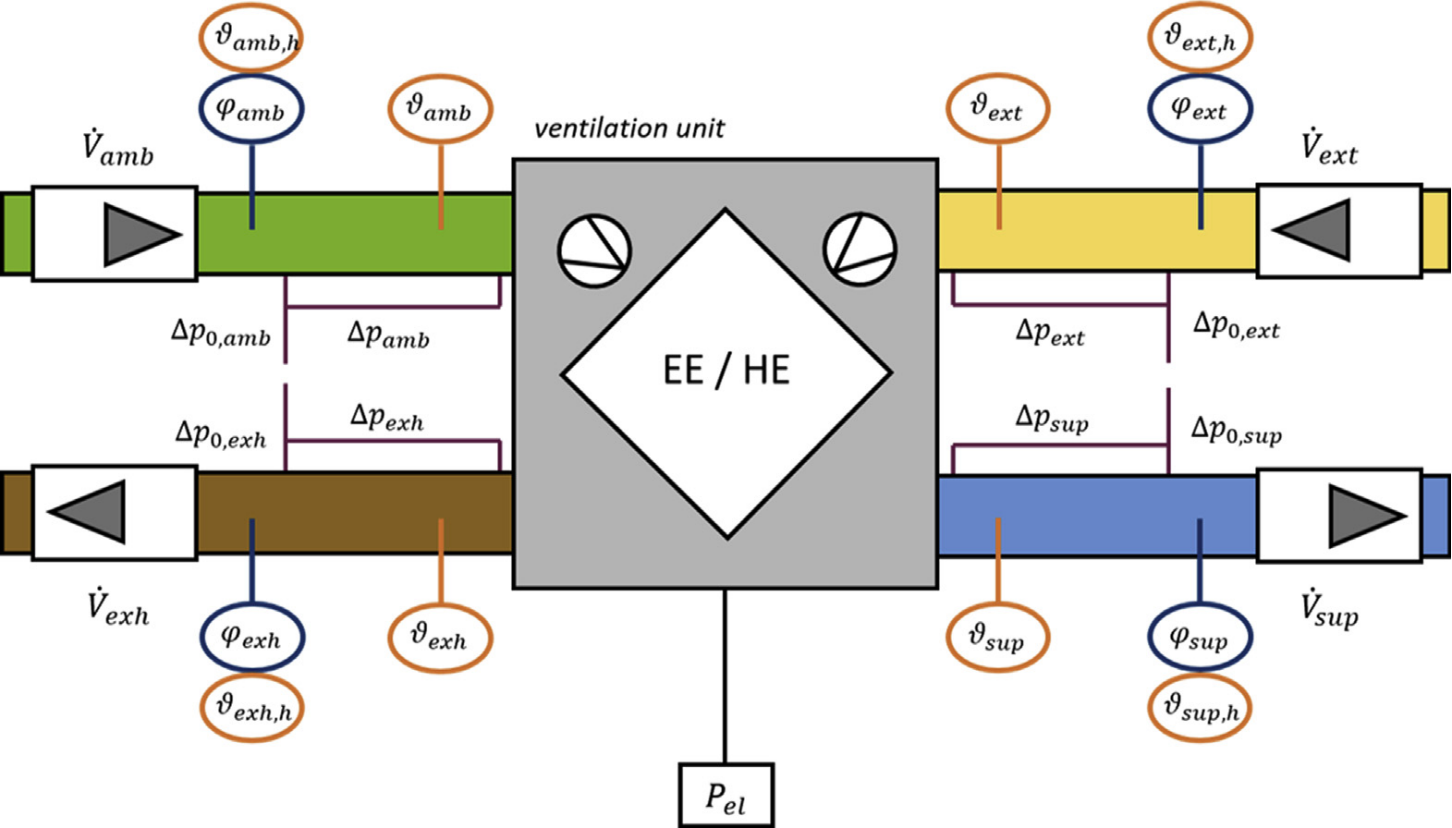
Schematic of the test rig for ventilation units.

In addition, the data of the equivalent air layer thickness of the enthalpy exchanger's membrane can be found as figure in the data (see Fig. 2). The experimental set-up for the determination of these data can be found in Ref. [1] (see Fig. 3).

## 2 Experimental design, materials, and methods

### 2.1 Experimental set-up

Fig. 2 shows the experimental set-up which is used in a test rig for ventilation units. Instead of a whole ventilation unit in this case only the exchanger was measured. For this reason no information about the electrical power (${\text{P}}_{\text{el}}$) is delivered, because the volume flow was established with external ventilators. The pipes are connected to two climate chambers (not shown here). One climate chamber emulates the room air and the second one emulates the ambient air. Both climate chambers can be controlled with respect to temperature (heating and cooling) and moisture (humidification and de-humidification). The heat exchanger was situated in a third room with a constant room temperature (equal to the extract air temperature of the heat exchanger). The temperature at the inlets can be kept constant within ±0.05°C and the relative humidity can be maintained within ±2.0% during a measurement cycle. For both air streams, the conditions at the inlet and outlet are measured. The temperature is measured near the inlet to the heat exchanger with five temperature sensors (ϑ). Additionally, the relative humidity (φ) and the temperature are measured within a short distance (to ensure uniform air properties by the mixing of the air). The volume flow $\dot{V}$ is measured with orifice plates and differential pressure indicators. Depending on the range of the expected volume flow, different orifice plates are used. Oversized running-in sections guarantee a uniform flow. In addition the differential pressure (Δp) between the inlets and outlets of the heat exchanger was measured.

All air ducts used to connect the ventilation unit to the test rig were insulated with a 13 cm thick layer (Armaflex and rock wool) including all pipes and the volume flow measurement. The exchanger unit itself was packed in a polystyrene case (approx. 2.5 cm thickness) with additional 3 cm of insulation.

### 2.2 Calibration and measurement inaccuracy

Table 1 shows the measurement devices used and the accuracies that can be achieved for each of it. The measurement inaccuracy cannot be assumed to be constant therefore for the whole data set the inaccuracies are additionally provided.

**Table 1:** Measurement devices and accuracy.

Measurement value	Position	SI unit	Measurement device	Measurement accuracy
Temperature	$\vartheta_{amb}$	°C	1x Pt100 4-wire with NI 9217 4x thermocouples type K with NI 9213	Pt100: ±0,05°C TC: ±0,10°C
	$\vartheta_{ext}$			
	$\vartheta_{exh}$			
	$\vartheta_{sup}$			
Relative humidity	$\vartheta_{amb,h}$	°C%	E + E EE210 Pt100 and capacitive humidity sensors	ϑ:±0,10°Cφ:±1,5%
	$\vartheta_{ext,h}$			
	$\vartheta_{exh,h}$			
	$\vartheta_{sup,h}$			
	$\varphi_{amb}$			
	$\varphi_{ext}$			
	$\varphi_{exh}$			
	$\varphi_{sup}$			
Humidity ratio	$x_{amb}$	$\frac{\text{g}}{\text{kg}}$	Derived from relative humidity and temperature	@ 21°C, 50%: ±0,24g/kg @ 0°C, 60%: ±0,06g/kg
	$x_{ext}$			
	$x_{exh}$			
	$x_{sup}$			
Enthalpy	$h_{amb}$	$\frac{\text{kJ}}{\text{kg}}$	Derived from absolute humidity and temperature	@ 21°C, 50%: ±0,87kJ/kg @ 4°C, 60%: ±0.36kJ/kg
	$h_{ext}$			
	$h_{exh}$			
	$h_{sup}$			
Volume flow	${\dot{V}}_{amb}$	$\frac{\text{m}³}{\text{h}}$	FläktWoods MR Huba Control 699	±5.0%
	${\dot{V}}_{ext}$			
	${\dot{V}}_{exh}$			
	${\dot{V}}_{sup}$			
Mass flow	${\dot{m}}_{amb}$	$\frac{\text{kg}}{\text{s}}$	Derived from volume flow, absolute humidity and temperature	±5.0%
	${\dot{m}}_{ext}$			
	${\dot{m}}_{exh}$			
	${\dot{m}}_{sup}$			
Absolute pressure	$p_{abs}$	Pa	THIES Clima Barogeber	±25 Pa
Differential pressure	$\text{Δ}p_{amb-sup}$	Pa	Huba Control 699	±2 Pa
	$\text{Δ}p_{ext-exh}$			
	$\text{Δ}p_{0,amb}$			
	$\text{Δ}p_{0,ext}$			
	$\text{Δ}p_{0,exh}$			
	$\text{Δ}p_{0,sup}$			
Electrical power consumption	$P_{el}$	W	Iskra MT 400	±0.5%
Temperature recovery effectiveness	$\eta_{T,1}$	%	exhaust air side, derived from temperature	@ $\vartheta_{ext}=21\;{}^{\circ}C$, $\vartheta_{amb}=4\;{}^{\circ}C$±0.5%
Temperature recovery effectiveness	$\eta_{T,2}$	%	supply air side, derived from temperature	@ $\vartheta_{ext}=21\;{}^{\circ}C$, $\vartheta_{amb}=4\;{}^{\circ}C$±0.5%
Moisture recovery effectiveness	$\eta_{x,exh}$	%	exhaust air side, derived from absolute humidity	@ $\vartheta_{ext}=21\;{}^{\circ}C$, $\vartheta_{amb}=4{}^{\circ}C$, $\varphi_{ext}=50\%$±3.8%
Moisture recovery effectiveness	$\eta_{x,sup}$	%	supply air side, derived from absolute humidity	@ $\vartheta_{ext}=21\;{}^{\circ}C$, $\vartheta_{amb}=4\;{}^{\circ}C$, $\varphi_{ext}=50\;\%$±3.2%

The calibration of the temperature sensors (Pt100 and thermocouple) is done with an internal calibration equipment based on a calibration bath and two reference sensors (Dostmann T4200 and Burster KELVIMAT 4306) which itself are calibrated according to ISO standards. The calibration was done in a temperature range of −10 °C–50 °C with 1 K steps.

The calibration of the humidity sensors is done with a humidity calibrator (Michell Instruments HygroCal100) and a dew point mirror (Michell Instruments Optidew) as reference. The calibration was done between 10% and 90% relative humidity (in steps of 10%) at a room temperature of around 5 °C, 15 °C and 25 °C.

For the calibration of the volume flow a WÖHLER DP 700 is used. It uses a laminar flow element and a hot-film anemometer. The calibration was done for a temperature range of the air flow between 5 °C and 35 °C.

### 2.3 Boundary conditions

Table 2 and Table 3 show the boundary conditions during the measurement, which are typically winter and summer conditions called.

**Table 2:** Winter conditions.

Parameter	Heat Exchanger	Enthalpy Exchanger
Volume flow ${\dot{V}}_{sup}={\dot{V}}_{ext}$	80, 110, 150 m³/h	80, 110, 150 m³/h
Ambient air temperature $\vartheta_{amb}$	−4, 0, 4, 7, 12 °C	−7, −4, 0, 4, 7, 12 °C
Extract air temperature $\vartheta_{ext}$	21 °C	21 °C
Ambient air relative humidity $\varphi_{amb}$	not conditioned	not conditioned
Extract air relative humidity $\varphi_{ext}$	no condensation in HE	20%–60%

**Table 3:** Summer conditions.

Parameter	Heat Exchanger	Enthalpy Exchanger
Volume flow ${\dot{V}}_{sup}={\dot{V}}_{ext}$	not investigated	80, 110, 150 m³/h
Ambient air temperature $\vartheta_{amb}$		25, 30 °C
Extract air temperature $\vartheta_{ext}$		25 °C
Ambient air relative humidity $\varphi_{amb}$		70%–90%
Extract air relative humidity $\varphi_{ext}$		60%–70%

### 2.4 Used efficiency parameters

For evaluating heat and enthalpy exchangers the method of effectiveness (for temperature and moisture) is used (compare (1) to (4)). Please be aware of the measurement uncertainties, especially in case of using $\eta_{x}$.

(1) $$\eta_{T,exh}=\frac{\vartheta_{ext}-\vartheta_{exh}}{\vartheta_{ext}-\vartheta_{amb}}$$

(2) $$\eta_{T,sup}=\frac{\vartheta_{sup}-\vartheta_{amb}}{\vartheta_{ext}-\vartheta_{amb}}$$

(3) $$\eta_{x,exh}=\frac{x_{ext}-x_{exh}}{x_{ext}-x_{amb}}$$

(4) $$\eta_{x,sup}=\frac{x_{sup}-x_{amb}}{x_{ext}-x_{amb}}$$
